# Associations of the Geriatric Nutritional Risk Index With Femur Bone Mineral Density and Osteoporosis in American Postmenopausal Women: Data From the National Health and Nutrition Examination Survey

**DOI:** 10.3389/fnut.2022.860693

**Published:** 2022-05-17

**Authors:** Jie Wang, Fei Xing, Ning Sheng, Zhou Xiang

**Affiliations:** Department of Orthopedics, Orthopedic Research Institute, West China Hospital, Sichuan University, Chengdu, China

**Keywords:** geriatric nutrition risk index, nutrition, bone mineral density, osteoporosis, American postmenopausal women

## Abstract

**Background:**

The geriatric nutritional risk index (GNRI) has been used as a significant tool to access the nutritional status of the elderly. However, the relationship between the GNRI and femur bone mineral density (BMD) and the risk of osteoporosis remains unclear in American postmenopausal women.

**Objectives:**

We aimed to explore associations between the GNRI with femur BMD and the risk of osteoporosis in American postmenopausal women.

**Methods:**

We merged the continuous National Health and Nutrition Examination Survey (NHANES) 2005–2006, 2007–2008, 2009–2010, 2013–2014, and 2017–2018 to ensure a large and representative sample, including 3,152 participants. The linear relationship between the GNRI and femur BMD was assessed *via* a weighted multivariate linear regression model. The odds ratios (ORs) and 95% confidence intervals (95% CIs) for the association between the GNRI and the risk of osteoporosis were assessed by a weighted logistic regression model. Moreover, the nonlinear relationship was also characterized by smooth curve fitting (SCF) and a weighted generalized additive model (GAM).

**Results:**

After adjusting for all covariates, the weighted multivariable linear regression models demonstrated that the GNRI was positively correlated with femur BMD. The weighted logistic regression models demonstrated that each unit of increased GNRI value was associated with a decreased risk of osteoporosis of 4.13%. When categorizing GNRI based on quartiles, ORs between the risk of osteoporosis and the GNRI across quintiles 2, 3, and 4 compared with quintile 1 were 0.5565 (95% CI: 0.4791, 0.6463; *P* < 0.000001), 0.5580 (95% CI: 0.4600, 0.6769; *P* < 0.000001), and 0.3475 (95% CI: 0.2681, 0.4505; *P* < 0.000001). The trends similar to the above were also observed in SCF and GAM.

**Conclusion:**

This study indicated that nutritional status, represented by the GNRI, was positively associated with femur BMD and negatively associated with the risk of osteoporosis in American postmenopausal women. The GNRI may be a good tool to identify American postmenopausal women who need further bone health nutritional support.

## Introduction

Osteoporosis is a kind of skeletal disease with degradation of bone tissue microstructure and low bone mineral density (BMD). It usually results in increased bone fragility and an increased risk of fractures (1). In the United States, there are 1.5 million fractures caused by osteoporosis each year, most of which occur in postmenopausal women (2). This can lead to a poor quality of life, a dependent living situation, increased fracture-related mortality, and medical care costs. Especially in elderly women, hip fractures can be devastating (2). Therefore, the prevention and management of low femur BMD and osteoporosis in postmenopausal women are of great significance (3, 4). In addition, identifying the disease-related risk factors is a clinical priority.

Several studies have reported that nutrients, including some micronutrients such as calcium, magnesium, phosphorous, and vitamin D, could influence BMD (5, 6). Besides, an easily neglected fact is that proteins are also crucial for bone health. Adequate protein intake contributes to bone development and bone maintenance (7). As key constituents of the bone mineral matrix, proteins regulate bone metabolism by providing building blocks and performing specific regulatory functions (8). In recent years, the geriatric nutritional risk index (GNRI) has been used as a significant tool to access the nutritional status of the elderly. It reflects the level of serum albumin and also includes height and body weight for the overall evaluation (9). Some studies have shown that the GNRI is associated with nutritional-related complications in hospitalized elderly patients (10), patients with hemodialysis (11), patients with chronic kidney disease (12), patients with heart failure (13), and patients with obstructive pulmonary disease (14).

However, the relationship between the GNRI and femur BMD and the risk of osteoporosis has not been adequately investigated. To the best of our knowledge, no studies have evaluated this relationship in American postmenopausal women (15, 16). The aim of this study was to explore associations of the GNRI with femur BMD and the risk of osteoporosis in this specific population.

## Materials and Methods

### Study Population

**FIGURE 1:**
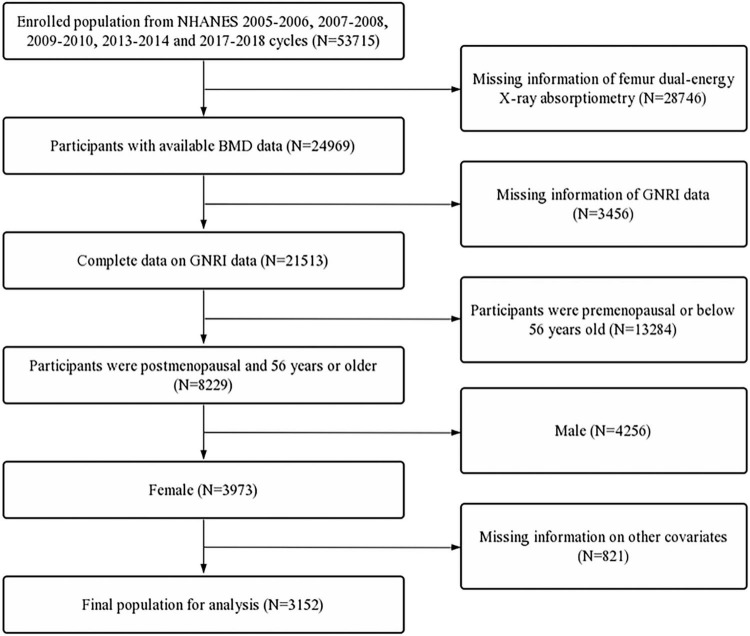
Flow diagram of the inclusion criteria and exclusion criteria. NHANES, National Health and Nutrition Examination Survey; GNRI, geriatric nutritional risk index; BMD, bone mineral density.

Data used in this study were extracted from the National Health and Nutrition Examination Survey (NHANES). NHANES data were collected from a nationally representative sample of American civilians using a multistage probability design. All participants provided written informed consent, and NHANES was approved by the National Center for Health Statistics Ethics Review Board. For the study, we merged the continuous NHANES 2005–2006, 2007–2008, 2009–2010, 2013–2014, and 2017–2018 to ensure a large and representative sample. The inclusion criteria were as follows: (1) participants with available femur BMD and GNRI data, and (2) postmenopausal women and women over the age of 55. The exclusion criteria were participants with incomplete data on race/ethnicity, educational level, marital status, poverty income ratio (PIR), body mass index (BMI), who smoked at least 100 cigarettes, with hypertension status, with diabetes status, who ever used prednisone or cortisone daily, who ever used female hormones, who had a hysterectomy, with moderate or vigorous activity, and with a postmenopausal period (Figure 1).

### BMD and Definition of Osteoporosis

Bone mineral density was evaluated using dual-energy x-ray absorptiometry scans with Hologic QDR-4500A fan-beam densitometers (Hologic, Inc., Bedford, MA, United States). The assessed femoral regions included total femur, femur neck, trochanter, and intertrochanter. According to the World Health Organization classification criteria, a BMD value in any femoral region lower than –2.5 standard deviations of the young adult reference group can be defined as osteoporosis. The specific thresholds were 0.68 g/cm^2^, 0.59 g/cm^2^, 0.49 g/cm^2^, and 0.78 g/cm^2^ for total femur, femur neck, trochanter, and intertrochanter, respectively (17).

### Geriatric Nutritional Risk Index

According to the parameters of serum albumin (g/L), ideal body weight (WL0; kg), and actual body weight (kg), the GNRI was calculated as follows (18):


$$\text{GNRI}=\left(1.489\times \mathrm{albumin}\right)+\left(41.7\times \frac{\text{weight}}{\mathrm{WL0}}\right)$$

The WL0 can be calculated using the parameter of height (H; cm) as follows:


$$\mathrm{Women}:\mathrm{WL0}=\text{H}-100-\left(\frac{\left[H-150\right]}{2.5}\right)$$

### Covariates

Based on the previous literature and clinical experience, the selected covariates were obtained as follows:

1. Demographic data: age (66–70 years, ≥70 years), race/ethnicity (Mexican Americans, other Hispanic, non-Hispanic white, non-Hispanic black, and other races), educational level (less than 9th grade, 9–11th grade, high school, some college, and college graduate), marital status (married, widowed, divorced, separated, never married, and living with a partner), and PIR (≤ 1, 1–3, and > 3).
2. Examination data: BMI (< 25, 25–30, and > 30).
3. Questionnaire data: smoked at least 100 cigarettes (yes or no), hypertension status (yes or no), diabetes status (yes, no, or borderline), ever used prednisone or cortisone daily (yes or no), ever used female hormones (yes or no), had a hysterectomy (yes or no), moderate or vigorous activity (yes or no), which was defined by the criterion that usually does moderate or vigorous activities for at least 10 min that cause a moderate or vigorous increase in breathing or heart rate, and postmenopausal period, which was calculated by the age when taking the questionnaire minus age at last menstrual period.

### Statistical Analysis

Based on the weight selection criteria of NHANES, the weight value used in the study was one-fifth of the full sample two-year mobile examination center of exam weight. Data from continuous and categorical variables were described by the mean and proportion, respectively. The Chi-squared test was used to compare the differences in categorical variables between the osteoporosis and non-osteoporosis groups and for continuous variables, a Student’s *t*-test was used. The linear relationship between the GNRI and femur BMD was assessed using a weighted multivariate linear regression model. The odds ratios (ORs) and 95% confidence intervals (95% CIs) for the association between the GNRI and the risk of osteoporosis were assessed using a weighted logistic regression model. Model 1 was adjusted for no covariates. Model 2 was adjusted for age, race, and BMI. Model 3 was adjusted for all the covariates. Moreover, we performed subgroup analyses using weighted stratified line regression models based on age. The nonlinear relationship in this study was also characterized by smooth curve fitting (SCF) and a weighted generalized additive model (GAM). Furthermore, the following analyses were performed to ensure the robustness of the data analysis. First, the values of GNRI were categorized based on quartiles, and tests for linear trends were performed. All the steps described above were also performed to evaluate the relationship between the categorized GNRI and femur BMD and the risk of osteoporosis.

All analyses were performed using R software (4.0.3) and EmpowerStats (2.0). A two-sided *p-*value < 0.05 was considered to have statistical significance.

## Results

### Baseline Characteristics of Participants

First, 53,715 participants were selected from the NHANES 2005–2006, 2007–2008, 2009–2010, 2013–2014, and 2017–2018. In these datasets, participants with missing femur BMD data (*n* = 28,746) and incomplete GNRI data (*n* = 3,456) were excluded. Furthermore, participants aged below 56 years (*n* = 13,284), male participants (*n* = 4,256), and participants with missing data on other covariates (*n* = 821) were also excluded. A total of 3,152 participants were included in the final analysis (Figure 1).

The baseline characteristics of selected participants were compared between the osteoporosis and non-osteoporosis groups (Table 1). According to the diagnosis criteria for osteoporosis (17), the prevalence of osteoporosis was 21.200% (668/3,152) in this study. Compared with patients with osteoporosis, participants without osteoporosis were more likely to have higher values of GNRI (125.122 ± 12.739 vs. 116.510 ± 11.406, *P* < 0.00001) but shorter postmenopausal years (18.882 ± 10.954 vs. 24.791 ± 11.544, *P* < 0.00001). Moreover, participants in the osteoporosis group tended to be older, more emaciated, widowed, smoked more cigarettes, poorer, have less activity, and have lower educational levels. Besides, the percentage of participants who ever used female hormones and had a hysterectomy was significantly higher in the osteoporosis group (*P* < 0.05, Table 1).

**TABLE 1:** Weighted characteristics of the study population.

	Non-osteoporosis (*N* = 2484, 78.807%)	Osteoporosis (*N* = 668, 21.200%)	*P* value
GNRI (mean ± SD)	125.122 ± 12.739	116.510 ± 11.406	< 0.00001
Age (%)			< 0.00001
<70	69.628	46.018	
≥70	30.372	53.982	
Race (%)			0.00004
Mexican Americans	4.076	2.84	
Other Hispanic	2.941	3.577	
Non-Hispanic White	79.618	82.341	
Non-Hispanic Black	8.648	3.893	
Other race	4.716	7.349	
BMI (%)			< 0.00001
<25	24.751	50.814	
≥25, <30	34.068	32.603	
≥30	41.181	16.583	
PIR (%)			< 0.00001
<1	7.914	9.48	
≥1, <3	37.851	49.126	
≥3	54.235	41.393	
Educational level (%)			< 0.00001
Less than 9th grade	4.502	7.255	
9–11th grade	9.366	13.209	
High school	27.746	29.845	
Some college	29.79	29.606	
College graduate	28.596	20.085	
Marital status (%)			< 0.00001
Married	58.725	45.263	
Widowed	18.15	33.207	
Divorced	16.354	16.737	
Separated	1.373	0.913	
Never married	3.638	2.776	
Living with partner	1.761	1.105	
Diabetes status (%)			0.17031
Yes	14.696	12.555	
No	82.035	85.04	
Borderline	3.27	2.405	
Hypertension status (%)			0.4737
Yes	55.32	53.765	
No	44.68	46.235	
Ever use prednisone or cortisone daily (%)			0.73002
Yes	8.013	8.423	
No	91.987	91.577	
Smoked at least 100 cigarettes (%)			0.0036
Yes	39.548	45.789	
No	60.452	54.211	
Had a hysterectomy (%)			0.00245
Yes	40.603	47.124	
No	59.397	52.876	
Ever use female hormones (%)			0.00006
Yes	49.633	40.903	
No	50.367	59.097	
Postmenopausal period (years, mean ± SD)	18.882 ± 10.954	24.791 ± 11.544	< 0.00001
Moderate or vigorous activity (%)			0.00165
Yes	51.054	44.194	
No	48.946	55.806	
Total femur BMD (g/cm^2^, mean ± SD)	0.882 ± 0.111	0.669 ± 0.076	< 0.00001
Femur neck BMD (g/cm^2^, mean ± SD)	0.741 ± 0.103	0.550 ± 0.070	< 0.00001
Trochanter BMD (g/cm^2^, mean ± SD)	0.666 ± 0.097	0.508 ± 0.079	< 0.00001
Intertrochanter BMD (g/cm^2^, mean ± SD)	1.051 ± 0.139	0.800 ± 0.106	< 0.00001

*BMD, bone mineral density; GNRI, geriatric nutritional risk index; PIR, poverty income ratio; BMI, body mass index; SD standard deviation; %, weighted percentage.*

### Associations of GNRI With Femur BMD and Osteoporosis

**FIGURE 2:**
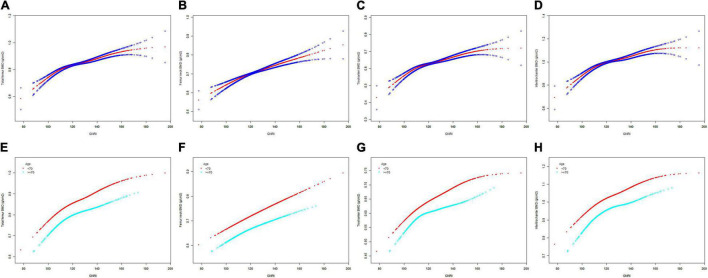
The SCF for the association between GNRI and femur BMD. Age [in panels **(A–D)**], race/ethnicity, BMI, educational level, marital status, PIR, smoked at least 100 cigarettes, hypertension status, diabetes status, ever used prednisone or cortisone daily, ever used female hormones, had a hysterectomy, moderate or vigorous activity, and postmenopausal period were adjusted. **(A,E)** Total femur BMD; **(B,F)** Femur neck BMD; **(C,G)** Trochanter BMD; **(D,H)** Intertrochanter BMD. SCF, smooth curve fit; BMD, bone mineral density; GNRI, geriatric nutritional risk index; PIR, poverty income ratio; BMI, body mass index.

**FIGURE 3:**
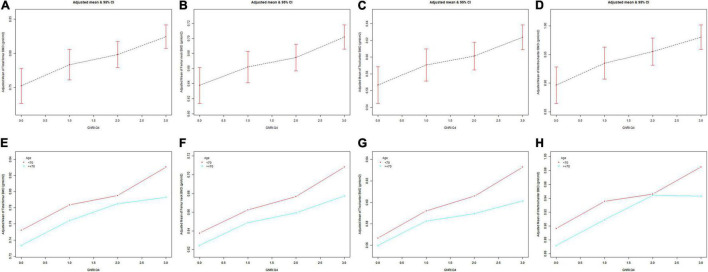
The association between GNRI.Q4 and femur BMD. Age [in panels **(A–D)**], race/ethnicity, BMI, educational level, marital status, PIR, smoked at least 100 cigarettes, hypertension status, diabetes status, ever used prednisone or cortisone daily, ever used female hormones, had a hysterectomy, moderate or vigorous activity, and postmenopausal period were adjusted. **(A,E)** Total femur BMD; **(B,F)** Femur neck BMD; **(C,G)** Trochanter BMD; **(D,H)** Intertrochanter BMD. BMD, bone mineral density; GNRI.Q4, geriatric nutritional risk index quartiles; PIR, poverty income ratio; BMI, body mass index.

**FIGURE 4:**
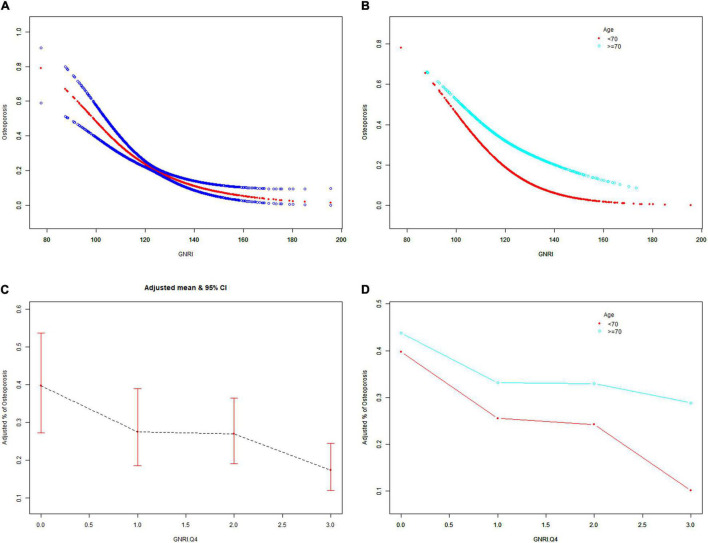
The associations of GNRI and GNRI.Q4 with the risk of osteoporosis. Age [**(A,C)** were applicable, **(B,D)** were not applicable], race/ethnicity, BMI, educational level, marital status, PIR, smoked at least 100 cigarettes, hypertension status, diabetes status, ever use prednisone or cortisone daily, ever use female hormones, had a hysterectomy, moderate or vigorous activity, and postmenopausal period were adjusted. BMD, bone mineral density; GNRI.Q4, geriatric nutritional risk index quartiles; PIR, poverty income ratio; BMI, body mass index.

The GNRI values showed a positive association with femur BMD and a negative association with the risk of osteoporosis in Model 1. After adjusting for confounding factors in Model 2 (age, race/ethnicity, and BMI) and Model 3 (age, race/ethnicity, BMI, educational level, marital status, PIR, smoked at least 100 cigarettes, hypertension status, diabetes status, ever used prednisone or cortisone daily, ever used female hormones, had a hysterectomy, moderate or vigorous activity, and postmenopausal period), the relationship between exposed variables and outcomes was still stable. When adjusting for all covariates, each unit of increased GNRI value was associated with a decreased risk of osteoporosis of 4.13% (Table 2). After categorizing GNRI based on quartiles, ORs between the risk of osteoporosis and GNRI values across quintiles 2, 3, and 4 compared with quintile 1 were 0.5565 (95% CI: 0.4791, 0.6463; *P* < 0.000001), 0.5580 (95% CI: 0.4600, 0.6769; *P* < 0.000001), and 0.3475 (95% CI: 0.2681, 0.4505; *P* < 0.000001), respectively, in Model 3. The trend test also showed that, with the increase of GNRI quartile groups, the risk of osteoporosis decreased (for trend, *P* < 0.001) (Table 3). Moreover, the trends similar to the above were also observed in SCF and GAM (Figures 2A–D, 3A–D, 4A,C).

**TABLE 2:** Associations of the GNRI with femur BMD and the risk of osteoporosis.

	Model 1 β (95% CI) *P* value	Model 2 β (95% CI) *P* value	Model 3 β (95% CI) *P* value
Total femur BMD (g/cm^2^)	0.0048 (0.0044, 0.0051) < 0.000001	0.0026 (0.0021, 0.0032) < 0.000001	0.0024 (0.0019, 0.0029) < 0.000001
Femur neck BMD (g/cm^2^)	0.0037 (0.0034, 0.0040) < 0.000001	0.0019 (0.0014, 0.0024) < 0.000001	0.0017 (0.0012, 0.0023) < 0.000001
Trochanter BMD (g/cm^2^)	0.0037 (0.0034, 0.0039) < 0.000001	0.0020 (0.0016, 0.0025) < 0.000001	0.0019 (0.0014, 0.0024) < 0.000001
Intertrochanter BMD (g/cm^2^)	0.0056 (0.0052, 0.0060) < 0.000001	0.0029 (0.0022, 0.0036) < 0.000001	0.0026 (0.0020, 0.0033) < 0.000001
Osteoporosis	0.9381 (0.9340, 0.9421) < 0.000001	0.9529 (0.9458, 0.9600) < 0.000001	0.9587 (0.9514, 0.9660) < 0.000001

*Model 1: no covariates were adjusted.*

*Model 2: age, race/ethnicity, and BMI were adjusted.*

*Model 3: age, race/ethnicity, BMI, educational level, marital status, PIR, smoked at least 100 cigarettes, hypertension status, diabetes status, ever used prednisone or cortisone daily, ever used female hormones, had a hysterectomy, moderate or vigorous activity, and postmenopausal period were adjusted.*

*BMD, bone mineral density; GNRI, geriatric nutritional risk index; PIR, poverty income ratio; BMI, body mass index.*

**TABLE 3:** Associations of the GNRI.Q4 with femur BMD and the risk of osteoporosis.

	Model 1 β (95% CI) *P* value	Model 2 β (95% CI) *P* value	Model 3 β (95% CI) *P* value
**Total femur BMD (g/cm^2^)**			
GNRI.Q4			
Q1 (77.5361–114.9444)	References	References	References
Q2 (114.9520–122.5324)	0.0522 (0.0402, 0.0641) < 0.000001	0.0300 (0.0153, 0.0447) 0.000066	0.0234 (0.0090, 0.0378) 0.001463
Q3 (122.5404–130.8391)	0.1008 (0.0887, 0.1129) < 0.000001	0.0443 (0.0263, 0.0624) 0.000002	0.0365 (0.0188, 0.0542) 0.000055
Q4 (130.8491–195.4885)	0.1560 (0.1440, 0.1680) < 0.000001	0.0643 (0.0427, 0.0859) < 0.000001	0.0574 (0.0362, 0.0786)
*P* for trend	< 0.001	<0.001	< 0.001
**Femur neck BMD (g/cm^2^)**			
GNRI.Q4			
Q1 (77.5361–114.9444)	References	References	References
Q2 (114.9520–122.5324)	0.0414 (0.0302, 0.0526) < 0.000001	0.0253 (0.0115, 0.0390) 0.000326	0.0212 (0.0076, 0.0348) 0.002323
Q3 (122.5404–130.8391)	0.0778 (0.0664, 0.0892) < 0.000001	0.0371 (0.0202, 0.0540) 0.000018	0.0312 (0.0145, 0.0480) 0.000254
Q4 (130.8491–195.4885)	0.1230 (0.1118, 0.1342) < 0.000001	0.0567 (0.0365, 0.0769) < 0.000001	0.0506 (0.0305, 0.0706)
P for trend	< 0.001	<0.001	< 0.001
**Trochanter BMD (g/cm^2^)**			
GNRI.Q4			
Q1 (77.5361–114.9444)	References	References	References
Q2 (114.9520–122.5324)	0.0440 (0.0340, 0.0541) < 0.000001	0.0286 (0.0160, 0.0411) 0.000009	0.0233 (0.0110, 0.0356) 0.000211
Q3 (122.5404–130.8391)	0.0796 (0.0694, 0.0899) < 0.000001	0.0387 (0.0233, 0.0542) < 0.000001	0.0321 (0.0170, 0.0473) 0.000032
Q4 (130.8491–195.4885)	0.1219 (0.1118, 0.1321) < 0.000001	0.0547 (0.0362, 0.0732) < 0.000001	0.0495 (0.0314, 0.0676)
P for trend	< 0.001	<0.001	< 0.001
**Intertrochanter BMD (g/cm^2^)**			
GNRI.Q4			
Q1 (77.5361–114.9444)	References	References	References
Q2 (114.9520–122.5324)	0.0608 (0.0460, 0.0757) < 0.000001	0.0327 (0.0143, 0.0511) 0.000502	0.0243 (0.0062, 0.0424) 0.008461
Q3 (122.5404–130.8391)	0.1207 (0.1056, 0.1358) < 0.000001	0.0506 (0.0280, 0.0731) 0.000012	0.0408 (0.0186, 0.0630) 0.000325
Q4 (130.8491–195.4885)	0.1822 (0.1673, 0.1971) < 0.000001	0.0695 (0.0425, 0.0965) < 0.000001	0.0606 (0.0340, 0.0872) 0.000008
P for trend	< 0.001	<0.001	< 0.001
**Osteoporosis**			
GNRI.Q4			
Q1 (77.5361–114.9444)	1	1	1
Q2 (114.9520–122.5324)	0.4358 (0.3896, 0.4875) < 0.000001	0.5140 (0.4448, 0.5939) < 0.000001	0.5565 (0.4791, 0.6463)
Q3 (122.5404–130.8391)	0.3089 (0.2733, 0.3492) < 0.000001	0.4839 (0.4017, 0.5830) < 0.000001	0.5580 (0.4600, 0.6769)
Q4 (130.8491–195.4885)	0.1455 (0.1255, 0.1688) < 0.000001	0.3196 (0.2483, 0.4114) < 0.000001	0.3475 (0.2681, 0.4505)
P for trend	< 0.001	<0.001	< 0.001

*Model 1: no covariates were adjusted.*

*Model 2: age, race/ethnicity, and BMI were adjusted.*

*Model 3: age, race/ethnicity, BMI, educational level, marital status, PIR, smoked at least 100 cigarettes, hypertension status, diabetes status, ever used prednisone or cortisone daily, ever used female hormones, had a hysterectomy, moderate or vigorous activity, and postmenopausal period were adjusted.*

*BMD, bone mineral density; GNRI.Q4, geriatric nutritional risk index quartile; PIR, poverty income ratio; BMI, body mass index.*

### Subgroup Analyses

After stratification by age, the results presented a similar trend to the above. Whether or not the participants were older than 70 years, the GNRI values presented a positive association with femur BMD and a negative association with the risk of osteoporosis in Model 1, Model 2, and Model 3. When adjusting for all covariates except age, each unit of increased GNRI value was associated with 2.74 and 4.64% decreased risk of osteoporosis in people aged below 70 years and those aged 70 years or older, respectively (Table 4). Moreover, in people aged below 70 years, the ORs between the risk of osteoporosis and GNRI across quintiles 2, 3, and 4 compared with quintile 1 were 0.6400 (95% CI: 0.5193, 0.7887; *P* = 0.000028), 0.7007 (95% CI: 0.5321, 0.9229; *P* = 0.011359), and 0.4918 (95% CI: 0.3274, 0.7387; *P* = 0.000628), respectively. In people who were 70 years or older, the ORs were 0.5055 (95% CI: 0.4074, 0.6273; *P* < 0.000001), 0.4877 (95% CI: 0.3702, 0.6426; *P* < 0.000001), and 0.3550 (95% CI: 0.2497, 0.5049; *P* < 0.000001), respectively (Table 5). In addition, the results of stratified analyses were also supported by the trends presented in SCF and GAM (Figures 2E–H, 3E–H, 4B,D).

**TABLE 4:** Associations of the GNRI with femur BMD and the risk of osteoporosis stratified by age.

	Age < 70 β (95% CI) *P* value	Age ≥ 70 β (95% CI) *P* value
**Total femur BMD (g/cm^2^)**		
Model 1	0.0044 (0.0040, 0.0048) < 0.000001	0.0048 (0.0042, 0.0053) < 0.000001
Model 2	0.0021 (0.0015, 0.0028) < 0.000001	0.0035 (0.0026, 0.0045) < 0.000001
Model 3	0.0019 (0.0012, 0.0026) < 0.000001	0.0032 (0.0023, 0.0042) < 0.000001
**Femur neck BMD (g/cm^2^)**		
Model 1	0.0035 (0.0031, 0.0038) < 0.000001	0.0034 (0.0029, 0.0040) < 0.000001
Model 2	0.0014 (0.0007, 0.0020) 0.000035	0.0029 (0.0020, 0.0037) < 0.000001
Model 3	0.0012 (0.0006, 0.0019) 0.000262	0.0025 (0.0017, 0.0034) < 0.000001
**Trochanter BMD (g/cm^2^)**		
Model 1	0.0035 (0.0032, 0.0039) < 0.000001	0.0034 (0.0029, 0.0039) < 0.000001
Model 2	0.0018 (0.0012, 0.0024) < 0.000001	0.0025 (0.0016, 0.0033) < 0.000001
Model 3	0.0016 (0.0011, 0.0022) < 0.000001	0.0022 (0.0014, 0.0031) < 0.000001
**Intertrochanter BMD (g/cm^2^)**		
Model 1	0.0050 (0.0045, 0.0055) < 0.000001	0.0059 (0.0052, 0.0066) < 0.000001
Model 2	0.0022 (0.0014, 0.0031) < 0.000001	0.0042 (0.0030, 0.0054) < 0.000001
Model 3	0.0020 (0.0011, 0.0028) 0.000004	0.0038 (0.0026, 0.0050) < 0.000001
**Osteoporosis**		
Model 1	0.9286 (0.9228, 0.9345) < 0.000001	0.9529 (0.9470, 0.9589) < 0.000001
Model 2	0.9582 (0.9480, 0.9686) < 0.000001	0.9516 (0.9418, 0.9616) < 0.000001
Model 3	0.9726 (0.9620, 0.9834) < 0.000001	0.9536 (0.9434, 0.9640) < 0.000001

*Model 1: no covariates were adjusted.*

*Model 2: race/ethnicity and BMI were adjusted.*

*Model 3: race/ethnicity, BMI, educational level, marital status, PIR, smoked at least 100 cigarettes, hypertension status, diabetes status, ever used prednisone or cortisone daily, ever used female hormones, had a hysterectomy, moderate or vigorous activity, and postmenopausal period were adjusted.*

*BMD, bone mineral density; GNRI, geriatric nutritional risk index; PIR, poverty income ratio; BMI, body mass index.*

**TABLE 5:** Associations of the GNRI.Q4 with femur BMD and the risk of osteoporosis stratified by age.

	Age < 70 β (95% CI) *P* value	Age ≥ 70 β (95% CI) *P* value
**Total femur BMD (g/cm^2^)**		
GNRI.Q4		
Q1 (77.5361–114.9444)	References	References
Q2 (114.9520–122.5324)	0.0104 (−0.0084, 0.0291) 0.279484	0.0428 (0.0202, 0.0653) 0.000214
Q3 (122.5404–130.8391)	0.0183 (−0.0048, 0.0414) 0.119757	0.0629 (0.0350, 0.0908) 0.000011
Q4 (130.8491–195.4885)	0.0409 (0.0134, 0.0683) 0.003552	0.0779 (0.0436, 0.1123) 0.000010
P trend	0.004	< 0.001
**Femur neck BMD (g/cm^2^)**		
GNRI.Q4		
Q1 (77.5361–114.9444)	References	References
Q2 (114.9520–122.5324)	0.0149 (−0.0033, 0.0332) 0.108267	0.0307 (0.0103, 0.0512) 0.003230
Q3 (122.5404-130.8391)	0.0227 (0.0003, 0.0451) 0.047516	0.0432 (0.0180, 0.0685) 0.000815
Q4 (130.8491–195.4885)	0.0346 (0.0079, 0.0613) 0.011097	0.0679 (0.0368, 0.0990) 0.000020
P trend	0.013	< 0.001
**Trochanter BMD (g/cm^2^)**		
GNRI.Q4		
Q1 (77.5361–114.9444)	References	References
Q2 (114.9520–122.5324)	0.0147 (−0.0014, 0.0308) 0.073631	0.0359 (0.0167, 0.0552) 0.000266
Q3 (122.5404–130.8391)	0.0252 (0.0054, 0.0449) 0.012837	0.0412 (0.0174, 0.0650) 0.000717
Q4 (130.8491–195.4885)	0.0432 (0.0196, 0.0667) 0.000334	0.0549 (0.0256, 0.0842) 0.000252
P trend	< 0.001	<0.001
**Intertrochanter BMD (g/cm^2^)**		
GNRI.Q4		
Q1 (77.5361–114.9444)	References	References
Q2 (114.9520–122.5324)	0.0077 (−0.0157, 0.0311) 0.517915	0.0488 (0.0204, 0.0772) 0.000790
Q3 (122.5404–130.8391)	0.0147 (−0.0141, 0.0435) 0.317872	0.0803 (0.0452, 0.1155) 0.000008
Q4 (130.8491–195.4885)	0.0403 (0.0060, 0.0746) 0.021238	0.0883 (0.0451, 0.1315) 0.000067
P trend	0.021	< 0.001
**Osteoporosis**		
GNRI.Q4		
Q1 (77.5361–114.9444)	References	References
Q2 (114.9520–122.5324)	0.6400 (0.5193, 0.7887) 0.000028	0.5055 (0.4074, 0.6273) < 0.000001
Q3 (122.5404–130.8391)	0.7007 (0.5321, 0.9229) 0.011359	0.4877 (0.3702, 0.6426) < 0.000001
Q4 (130.8491–195.4885)	0.4918 (0.3274, 0.7387) 0.000628	0.3550 (0.2497, 0.5049) < 0.000001
P trend	< 0.001	<0.001

*Model 1: no covariates were adjusted.*

*Model 2: race/ethnicity and BMI were adjusted.*

*Model 3: race/ethnicity, BMI, educational level, marital status, PIR, smoked at least 100 cigarettes, hypertension status, diabetes status, ever used prednisone or cortisone daily, ever used female hormones, had a hysterectomy, moderate or vigorous activity, and postmenopausal period were adjusted.*

*BMD, bone mineral density; GNRI.Q4, geriatric nutritional risk index quartile; PIR, poverty income ratio; BMI, body mass index.*

## Discussion

Based on the representative sample of American postmenopausal women in NHANES (2005–2006, 2007–2010, 2013–2014, and 2017–2018), this study found that the GNRI value was positively correlated to the femur BMD and negatively correlated to the risk of osteoporosis in this population. In addition, we demonstrated that the above associations were stable and not affected by age subgroups. To the best of our knowledge, this study is the first to explore the associations of the GNRI with femur BMD and the risk of osteoporosis in American postmenopausal women.

Previous studies have explored the relationship between protein intake and the risk of osteoporosis. For example, Looker et al. found that optimal protein intake could help to enhance BMD and prevent osteoporosis and fractures in postmenopausal women (19). Rizzoli et al. have systematically summarized a series of reviews and meta-analyses about the impact of protein intake on bone health, highlighting the key message that optimal maintenance of bone health in adults requires adequate supplies of dietary proteins (20). As a reflection of nutritional status with regards to protein, serum albumin can be associated with BMD. In a large cross-sectional observation of 21,121 patients, Afshinnia et al. reported an independent association of osteoporosis with low levels of serum albumin and long-term hypoalbuminemia (21) that supported the view of Coin et al. (22). Moreover, the association between BMI and BMD has also been shown in many studies. Tomlinson et al. hold the view that bones could benefit from combining high BMI with moderate-to-vigorous activities and an optimal diet (23). The study of Lloyd et al. found that higher BMI was conducive to increasing BMD (24). The views of Wang et al. (25) and Wu et al. (26) also supported this result. Compared with the individual variables of serum albumin or BMI, the GNRI combines serum albumin with body weight and height, which can be more comprehensive and effective for evaluating systemic nutritional status. Besides, in a receiver operating characteristic analysis for predicting osteoporosis, compared with serum albumin, BMI, and age, the GNRI had the largest area under the curve, indicating that the GNRI was a powerful indicator to improve the accuracy of diagnosis (27).

The specific mechanisms leading to the positive correlation between the GNRI and BMD may be multiple. On the one hand, dietary protein supplements can increase insulin-like growth factor I (IGF-I) (20, 28, 29) and decrease parathyroid hormone (PTH) (20, 30) and further reduce age-related BMD loss. On the other hand, optimal protein intake can help to resist loss of muscle and prevent sarcopenia in the elderly (31–33). Previous studies have demonstrated that, although many potential confounding factors were adjusted, the risk of BMD loss was still higher in the sarcopenic population (34–38). As is known to all, muscles can influence bones through secreting bone factors and exerting physical forces (39). Some molecules secreted by skeletal muscle, such as IGF-I, interleukin-6 (IL-6), IL-15, basic fibroblast growth factor, myostatin, and osteoglycin, have impacts on bone metabolism (40). Physical forces are usually produced by gravity, locomotion, or external devices (41). In this respect, the positive effect of high BMI on BMD has been recognized as a result of increased mechanical loading exerted on the skeleton (42). In short, the mechanism of the significant associations between GNRI and BMD and the risk of osteoporosis may be explained by an increase in IGF-I, a decrease in PTH, and resistance to muscle loss.

There are several strengths in this study. First, we used a large, nationally representative database, which was collected using standardized protocols to minimize possible bias. Second, we adequately controlled for confounders and assessed the difference in the association of the GNRI with femur BMD and the risk of osteoporosis in diverse populations by stratifying age. Third, we also categorized the GNRI by quartiles and performed tests for linear trends to ensure the robustness and accuracy of the data analyses. In addition, this study also has some potential limitations. First, because this study was a cross-sectional analysis, the evidence for a causal relationship may be insufficient. Second, the data collected from questionnaires and interviews may result in recall bias. Third, although we have adjusted some covariates, other unmeasured confounding factors may also lead to potential bias. In the future, more prospective studies need to be performed to confirm the results of this study.

## Conclusion

Our results indicated that nutritional status, represented by the GNRI, was positively associated with femur BMD and negatively associated with the risk of osteoporosis in American postmenopausal women. The GNRI may be a good tool to identify American postmenopausal women who need further bone health nutritional support.

## Data Availability Statement

Publicly available datasets were analyzed in this study. This data can be found here: https://www.cdc.gov/nchs/nhanes/.

## Author Contributions

JW, FX, and NS: conceptualization and investigation. JW: methodology, analysis, and writing the original draft. JW, FX, NS, and ZX: writing—review and editing. All authors contributed to the development of this manuscript, and they read and approved the final version.

## Conflict of Interest

The authors declare that the research was conducted in the absence of any commercial or financial relationships that could be construed as a potential conflict of interest.

## Publisher’s Note

All claims expressed in this article are solely those of the authors and do not necessarily represent those of their affiliated organizations, or those of the publisher, the editors and the reviewers. Any product that may be evaluated in this article, or claim that may be made by its manufacturer, is not guaranteed or endorsed by the publisher.
